# NGF stimulation alters the transcriptome and surface TrkB expression in axons of dorsal root ganglion neurons^☆^

**DOI:** 10.1016/j.ynpai.2025.100194

**Published:** 2025-08-05

**Authors:** Maximilian Koch, Manas Kshirsagar, Ankita Rawat, Abdolhossein Zare, Felicitas Schlott, Thorsten Bischler, Panagiota Arampatzi, Michael Briese, Michael Sendtner

**Affiliations:** a Institute of Clinical Neurobiology, University Hospital Würzburg, Würzburg, Germany; b Department of Neurology, University Hospital Würzburg, Würzburg, Germany; c Department of Anesthesiology, University Hospital Würzburg, Würzburg, Germany; d Core Unit Systems Medicine, University of Würzburg, Würzburg, Germany

**Keywords:** DRG neuron, Nerve growth factor, Axonal transcriptome, Interleukin

## Abstract

Nerve growth factor (NGF) is released after injury from macrophages and other cell types and induces an inflammatory response in neurons, characterized by local subcellular reactions and transcriptomic modulation. NGF-induced axonal transcriptome modulation may be crucial for pain initiation and maintenance. To explore these acute modulations, we cultured dorsal root ganglion neurons in microfluidic chambers and stimulated the axons with NGF. We found that axonal levels of the *Il7* transcript encoding interleukin-7 (IL-7) are increased after NGF stimulation, followed by IL-7 release from axons. In growth cones of sensory neurons, we also observed a reorganization of the ribosomal subunits 60S and 40S in response to NGF stimulation. In addition, a dynamic change in the spatio-temporal distribution of the Tropomyosin Kinase B (TrkB) receptor occurs at the plasma membrane of sensory neuron growth cones. TrkB is recruited from the endoplasmic reticulum (ER) leading to increased cell surface levels. De-novo synthesis of TrkB seems to be limited to somatic regions of sensory neurons. Thus, cytosolic mechanisms within distal regions of the sensory neurons may autonomously regulate signaling and translation in response to external NGF stimuli.

## Highlights

• Analysis of the axonal transcriptome of DRG neurons cultured in microfluidic chambers.
• NGF stimulation induces upregulation of the transcript encoding Interleukin-7 (IL-7) in axons and the release of IL-7.
• Increased translocation of TrkB to the cell surface following NGF stimulation.

## 1 Introduction

Chronic pain is characterized by persistent nociceptive changes that last beyond the recovery period necessary for wound healing (Treede et al., 2019). This involves complex crosstalk between cells of the nervous and the immune system, as a consequence of tissue injury but also as contribution to pathomechanisms underlying fibromyalgia, diabetes mellitus, and rheumatoid arthritis (Basbaum et al., 2009, Costigan et al., 2009). Chronic pain involves the sensitization of neurons residing in dorsal root ganglia (DRG) accompanied by transcriptomic and proteomic alterations. This can lead to an altered excitation threshold and increased response, interpreted as a painful sensation (Devor, 2009). While gene expression changes occurring in cell bodies of DRG neurons during pain signaling have been described in detail, even at the single cell level, much less is known about transcriptome alterations occurring in neurites and nerve terminals where these neurons interact with cells of the immune system (Renthal et al., 2020).

During tissue injury and local inflammation, nerve growth factor (NGF) is released from macrophages, fibroblasts, and immune cells. This induces pain signaling pathways in nociceptive neurons by activating Tropomyosin Kinase A (TrkA) receptors at nerve endings (Barker et al., 2020, Denk et al., 2017). NGF stimulates the local translation of cAMP-responsive element (CRE)-binding protein (CREB) in axons, which is then retrogradely transported to the nucleus where it activates gene expression programs (Cox et al., 2008). In addition, TrkA activation might induce local responses such as adaptive changes of the cytoskeleton or modulation of ion channel function (Lippoldt et al., 2013, McMahon, 1996, Papakonstanti et al., 2000, Tymanskyj et al., 2025, Yamaguchi et al., 2001). At high concentrations, NGF elicits painful sensations. For example, intraepidermal injection of NGF leads to localized thermal and mechanical hypersensitivity (Rukwied et al., 2010). In addition to enhanced clustering of ion channels at nociceptive nerve terminals, NGF-induced pain signaling involves activation of the p38 mitogen-activated protein kinase (MAPK) and extracellular regulated kinase 1/2 (ERK1/2) pathways and concomitant induction of nuclear gene expression programs such as upregulation of the transient receptor potential ion channel TrpV1 (Barker et al., 2020, Ji et al., 1999, Ji et al., 2002).

Axons of sensory neurons grow large distances from their cell bodies to their innervated targets, utilizing local RNA signaling mechanisms for reacting to diverse signals from nearby cells (La Peña et al., 2019). In this study, we investigated axonal transcriptome alterations of sensory neurons upon acute NGF stimulation. We observed dysregulation of many transcripts that modulate the activation of immune cells, including the transcript encoding Interleukin-7 (IL-7). A companion paper by Biesold et al. in this special issue demonstrates that IL-7 accumulates in wound areas in a pre-clinical model of laparotomy in mice, potentially contributing to neutrophil recruitment and activation at these sites. Together, this supports the notion that mechanisms regulating the levels of mRNAs and their translation locally represent an early response during pain induction (La Peña et al., 2019).

## 2 Materials and methods

### 2.1 Ethics statement

All experiments with mice in this study were conducted in accordance with the regulations of the federal law on animal protection, in agreement with and under the control of the local veterinary authority. The C57 BL/6 mice were bred and housed in the animal facility of the Institute of Clinical Neurobiology, with constant conditions of a 12 h day and night cycle at 20–22 °C and humidity of 55–65 %. Food and water were accessible ad libitum.

### 2.2 Cell culture

DRGs of C57BL/6 E13 embryos were prepared and collected in Hanks’ Balanced Salt Solution (HBSS) (Gibco, 14170-138), and digested with 0.1 % Trypsin (Worthington Biochemical, LS003707) in 300 μl HBSS for 30 min at 37 °C. The DRGs were mechanically triturated after adding 300 μl cold Neurobasal medium (Gibco, 21103049) containing 10 % horse serum (Gibco, 16050–122), 1 % Glutamax (Gibco, 35050061) and 2 % B27 Supplement (Gibco, 17504001) by pipetting them 6 times through a 1000 μl tip and 6 times through a 200 μl tip. The resulting cell suspension was preplated in 10 ml Neurobasal medium containing 10 % horse serum, 1 % Glutamax and 2 % B27 for 90 min in a 10 cm Nuncleon-coated dish (Thermo Fischer Scientific, 150350). The supernatant containing sensory neurons was then centrifuged for 8 min at 400×*g*. Cells were seeded on coverslips or in IND150 chambers (Xona Microfluidics) on 1.5H coverslips (24 × 50 mm; Marienfeld, LH25.2) coated with 1 mg/ml poly-L-lysine hydrobromide (PLL; Sigma-Aldrich, P2636) and 2.5 μg/ml laminin-111 (Thermo Fisher Scientific, 23017-015). 150,000–250,000 cells were seeded per chamber or 10,000 cells per coverslip and cultured at 37 °C, 5 % CO_2_ in Neurobasal medium containing 2 % horse serum, 1 % Glutamax and 2 % B27. The somatic compartment contained 10 ng/ml NGF 2.5S (Alomone Labs, N-100) and 5 ng/ml each of CNTF, GDNF (PeproTech, 450–10) and BDNF. The axonal compartment contained 40 ng/ml NGF and 5 ng/ml each of CNTF and BDNF, establishing an NGF gradient that guides neurite growth to the axonal compartment. 1 μM 5-Fluorodeoxyuridine (ACROS Organics™, 10124860) was included until day *in vitro* (DIV) 3.

For NGF deprivation and stimulation, the axonal compartment of sensory neurons cultured in compartmentalized microfluidic chambers was washed three times and cultured in Neurobasal medium as described above except with NGF at 2 ng/ml for 12 h in the axonal compartment. Thereafter, the NGF concentration in the axonal compartment was increased to 100 ng/ml for 2 h. For CNTF deprivation and stimulation, the axonal compartment was instead completely deprived of CNTF for 24 h before stimulating the axonal compartment with 100 ng/ml CNTF for 2 h.

### 2.3 Axonal supernatant precipitation

For precipitation of proteins from axonal supernatants, a modified version of the Trichloroacetic acid (TCA) precipitation protocol was used (Hutchings et al., 2024). For this purpose, 450 μl axonal supernatant was collected after NGF stimulation and then centrifuged at 1,000×*g* for 5 min. The supernatant was then transferred to fresh tubes and centrifuged again for 30 min at 10,000×*g*. After transferring the supernatant to fresh tubes, 60 μl of 100 % TCA was added. The samples were then precipitated on ice for 1 h and centrifuged for 30 min at 16,000×*g*. The supernatant was carefully aspirated, and pellets were dissolved in 2 × Laemmli buffer. The samples were then sonicated and heated at 95 °C for 5 min before being subjected to SDS-PAGE. Following transfer to a nitrocellulose membrane, the following antibodies were used for immunoblotting: rabbit anti-IL-7 (PeproTech, 500-P57), donkey anti-rabbit IgG horseradish peroxidase (Jackson ImmunoResearch, 711-035-152).

### 2.4 RNA purification

Compartments were washed with 200 μl RNase-free PBS once. Following PBS removal, 100 μl RNA extraction buffer (PicoPure™ RNA-Isolation Kit, Thermo Fischer Scientific, KIT0204) was added to the axonal compartment and pipetted up and down at least 5 times and then transferred to a test tube. Subsequently, 100 μl RNA extraction buffer was added to the somatic compartment and pipetted up and down at least 5 times before being transferred. Thereafter, axonal and somatic total RNA was purified with the PicoPure™ RNA-Isolation Kit. Genomic DNA was removed by treatment with RNase-free DNase (QIAGEN, 79254). The resulting purified RNA was stored at −80 °C until further processing.

### 2.5 RNA sequencing

RNA was isolated from the axonal and somatic compartments of DRG neurons. RNA integrity from NGF somata was evaluated on a 2100 Bioanalyzer with the RNA 6000 Pico kit (Agilent Technologies) whereas RNA from CNTF somata was analyzed using a 5200 Fragment Analyzer with the HS Total RNA 15 nt kit (Agilent Technologies). RNA from axons was used directly due to the low amount. cDNA libraries suitable for sequencing were prepared with the SMART-Seq® v4 Ultra® Low Input RNA Kit (Takara) according to the manufacturer’s instructions (1/4 vol for the somata and full volume for the axons). Respective treatment and control samples were processed in parallel. The PCR amplification was performed using 10 PCR cycles for soma samples and 16 PCR cycles for axon samples. Libraries were quantified by QubitTM dsDNA HS Assay Kit (3.0 Fluometer; Thermo Fisher Scientific) and quality was evaluated on a 2100 Bioanalyzer with the High Sensitivity DNA kit (Agilent Technologies). 0.5 ng of each library was subjected to a tagmentation-based protocol (Nextera XT, Illumina) using a quarter of the recommended reagent volumes. Libraries were quantified again by the QubitTM dsDNA HS Assay Kit and analyzed on a 2100 Bioanalyzer with High Sensitivity DNA kit (Agilent Technologies) for NGF or 5200 Fragment Analyzer with HS NGS Fragment 1–6000 bp kit (Agilent Technologies) for CNTF before pooling. Sequencing of pooled libraries, spiked with 1 % PhiX control library, was performed in single-end mode with 100 nt read length on the NextSeq 2000 platform (Illumina) with a P2 sequencing kit for the NGF and the CNTF samples, respectively. Demultiplexed FASTQ files were generated with bcl-convert (Illumina) v4.0.3 (NGF) or v4.2.4 (CNTF).

To assure high sequence quality, Illumina reads were quality- and adapter-trimmed via Cutadapt (Martin, 2011) (v2.5) using a cutoff Phred score of 20 in NextSeq mode, and reads without any remaining bases were discarded (parameters: −-nextseq-trim = 20 −m 1 −a CTGTCTCTTATACACATCT). Processed reads were subsequently mapped to the mouse genome (NCBI RefSeq assembly GCF_000001635.27/GRCm39) using STAR (Dobin et al., 2013) (v2.7.2b) with default parameters except for including transcript annotations from RefSeq annotation version 109 (NGF) or RS_2023_04 (CNTF) for GRCm39. This annotation was also used to generate read counts on exon level summarized for each gene via featureCounts (v1.6.4) from the Subread package (Liao et al., 2014). Multi-mapping and multi-overlapping reads were counted unstranded with a fractional count for each alignment and overlapping feature (parameters: −s 0 −t exon −M −O −-fraction). The count output was utilized to identify differentially expressed genes using DESeq2 (Love et al., 2014) (v1.24.0). Read counts were normalized by DESeq2 and fold-change shrinkage was conducted by setting the parameter betaPrior to TRUE. Differential expression was assumed at adjusted p-value after Benjamini-Hochberg correction (padj) < 0.05 and |log2FoldChange| ≥ 1. Gene ontology enrichment analysis was performed using DAVID (Huang et al., 2009, Sherman et al., 2022) for differentially expressed transcripts. As background, transcripts with a baseMean > 1 were used. BioVenn was applied for comparison of datasets (Hulsen et al., 2008). Figures have been created with SRPlot (Tang et al., 2023).

### 2.6 cDNA synthesis and qPCR analysis

cDNA synthesis was performed with random hexamers using the First Strand cDNA Synthesis Kit (Thermo Fischer Scientific, K1612). The resulting reaction mix was diluted 1:5 with water. The qPCR reactions were then set up with the Luminaris HiGreen qPCR Master Mix (Thermo Fisher Scientific, K0994), using a LightCycler 96 (Roche). The qPCR primers were designed with the help of the Primer3Plus online design tool (Untergasser et al., 2012). The primers were Gapdh fw: 5′-GCAAATTCAACGGCACA-3′, Gapdh rev: 5′-CACCAGTAGACTCCACGAC-3′, Il7 fw: 5′-CTGCTCGCAAGTTGAAGCAA-3′, Il7 rev: 5′-TCACCAGTGTTTGTGTGCCT-3′. All reactions were performed with two technical replicates, using an annealing temperature of 60 °C. The resulting Ct values were averaged before normalization. We then calculated the relative expression with the ΔΔCt method, using *Gapdh* as housekeeping transcript.

### 2.7 Surface TrkB staining assay

TrkB present at the plasma membrane was visualized using a live staining protocol adopted from (Hennlein et al., 2023). Sensory neurons were grown in microfluidic chambers on a Laminin 211 + 221 substrate. After axonal NGF deprivation to 2 ng/ml for 12 h, axons were stimulated with 100 ng/ml for 1 min, 10 min, 30 min, and a control with no stimulation. The microfluidic chambers attached to the glass coverslip were then transferred onto ice. Thereafter, the cells were incubated with rabbit anti-TrkB antibody (Cell Signaling, 80E3), diluted 1:200 in cold Neurobasal medium containing 2 % horse serum, 2 % B27 and 1 % Glutamax for 45 min on ice. Following three washes with cold PBS, the cells were incubated with donkey anti-rabbit Cy5 secondary antibody (1:400) diluted in cold NB culture medium for 45 min on ice. Lastly, the cells were washed three times with cold PBS before fixation with 4 % paraformaldehyde (PFA) on ice for 10 min. Following fixation, the cells were immunostained with anti-Synapsin1 antibody (Synaptic Systems, 106 011) using the following immunocytochemistry protocol.

### 2.8 Immunocytochemistry

Sensory neurons were fixed for 10 min with 4 % PFA and then washed twice with PBS (pH 7.4). Cells were permeabilized with 0.2 % Triton X-100 for 20 min, washed three times with PBS followed by blocking with 10 % donkey serum in PBST for 30 min. Subsequently, the cells were incubated in primary antibodies in blocking solution overnight at 4 °C. The next day, cells were washed three times with PBST, followed by incubation with the secondary antibodies diluted in PBST for 1 h at room temperature. This was again followed by three washes with PBST and incubation of the cells with DAPI for 5 min (5 µg/ml). Lastly, cells were washed twice with PBST and once with ultrapure water, before mounting with FluorSave Reagent (Merck, 345789) for imaging. Used antibodies and their dilution are listed in Supplement Table 1.

### 2.9 Imaging and quantification

Images were acquired on a standard Olympus Fluoview 1000 confocal microscope equipped with 10× (NA: 0.25), 20× (NA: 0.75), 40× (OIL, NA: 1.30) and 60× (OIL, NA: 1.35) objectives. Fluorescence excitation was achieved with 405, 473, 559, and 633  nm lasers. Structured illumination microscopy (SIM) imaging was performed on a Zeiss ELYRA S.1 microscope equipped with a Plan-Apochromat 63 × NA 1.40 immersion-oil based objective and with lasers conforming to a 405 nm diode (50 mW), a 488 nm OPSL (100 mW), a 561 nm OPSL (100 mW), and a 642 nm diode laser (150 mW). Immunofluorescence signals were quantified using mean gray values of images measured from the original files after simple thresholding using FIJI (Schindelin et al., 2012). For identifying co-clusters of RPL24-S6 ribosomal subunits, the JaCop plugin in FIJI was utilized for automated counting (Bolte and Cordelières, 2006). The co-localization was assessed by Pearson’s correlation coefficient. Data processing, statistical analysis, and visualization were performed using Python (v3.12) in a Jupyter Notebook environment, using associated open-source libraries such as NumPy, pandas, and matplotlib (Harris et al., 2020, Hunter, 2007, McKinney, 2010, Kluyver et al., 2016).

Analysis of expression of IL-7 in immunofluorescence images was done in a semi-automated manner. Stainings were performed as described in 2.8. Growth cones were manually annotated, based on the Nfh immunosignal, as regions of interest for all images as polygons in QuPath (Bankhead et al., 2017), https://qupath.github.io/). Regions of interest were exported as JSON-files and binary image segmentations were generated. For each region of interest, the brightness of the anti-IL-7 immunostaining was calculated per pixel. Sum of Intensity over all pixels of a growth cone was divided by the number of pixels. GraphPad Prism (GraphPad Software, San Diego, California USA) software was used to perform statistical analyses. Brightness of the images was adjusted to increase visibility.

### 2.10 Puromycylation proximity ligation assay (Puro-PLA)

Sensory neurons were grown for DIV 3 on laminin-211 + 221-coated glass coverslips. NGF was deprived to 2 ng/ml for 12 h followed by treatment with 100 ng/ml NGF for 10 mins, and 10 µg/ml puromycin (Sigma-Aldrich, P8833) for 8 min at 37 °C. In negative control experiments, puromycin and NGF were omitted. After the respective treatments, the cells were washed twice with pre-warmed HBSS, and then fixed using periodate-lysine-paraformaldehyde (PLP) for 10 min according to a previously published protocol (Salehi et al., 2023, Zare et al., 2024). After fixation, cells were washed and permeabilized for a proximity ligation assay (Sigma Aldrich, Duolink® Proximity Ligation Assay) using antibodies against puromycin (Sigma-Aldrich, MABE343; 1:200 dilution) and the N-terminus of TrkB (Cell Signaling, 80E3; 1:200 dilution).

### 2.11 Western blot

**Fig. 1:**
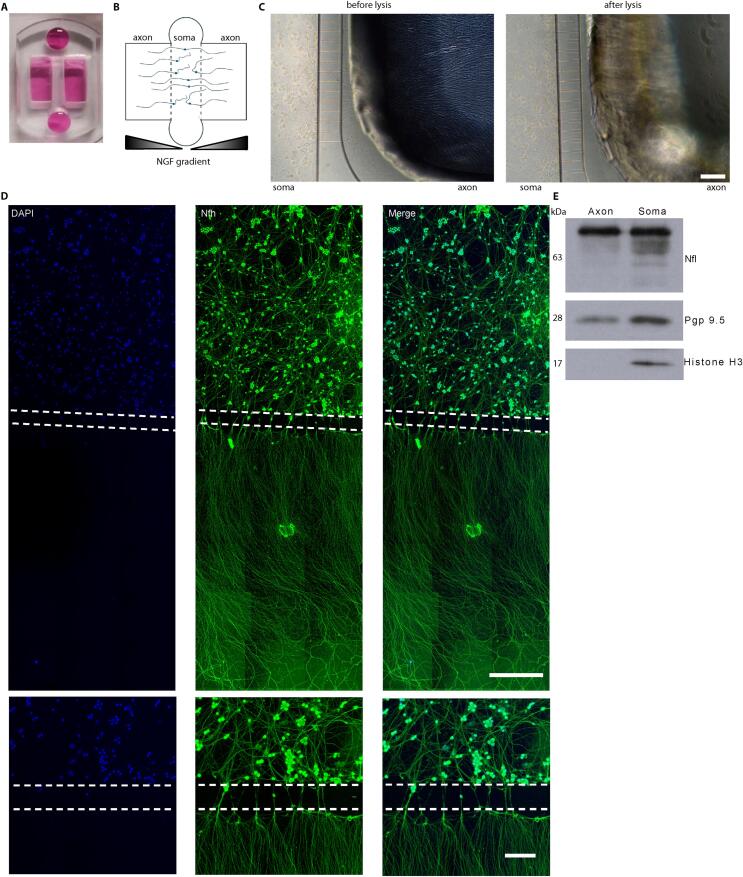
Compartmentalized cultures of embryonic DRG neurons. (A, B) Photograph (A) and schematic (B) of a microfluidic chamber containing a central somatic compartment and two outer axonal compartments. Application of an NGF gradient induces axonal growth towards the outer compartments. (C) Lysis of the axonal compartment for RNA extraction does not disturb the somatic compartment. (D) Immunofluorescence analysis of DRG neurons cultured in microfluidic chambers for DIV 4.5. Scale bars: 150 µm. (E) Immunoblot analysis of lysates extracted from the axonal and somatic compartment.

For protein extraction, the cells were washed with 200 μl PBS and then lysed with 50 μl Radioimmunoprecipitation assay (RIPA) buffer. The protein concentration was determined with a Bicinchoninic acid (BCA) assay (Thermo Fisher Scientific, 23227). 5 μl of sample were diluted with 45 µl of RIPA buffer and then mixed with 1 ml of the BCA mixture, which contained 50 parts BCA A, and one part BCA B. Samples were incubated at 37 °C for 30 min. The concentrations were determined by measuring them in a photometer, comparing the absorbance at 562 nm to a standard curve of protein concentration. 20 μg of protein were added per lane to a SDS gel. For the axonal supernatant, 30 μl were added per lane. The concentration of the resolving gel was 8 % for the plots in Fig. 1 E and 15 % for the IL-7 plots in Fig. 3 D, respectively.

Samples were mixed with 6x Laemmli buffer for an end concentration of 1x Laemmli and then boiled at 99 °C for 10 min. Gels were run at 80 V for 20 min and then at 120 V for up to 2 h, till the tracking dye was close to the edge of the gel or exited it. The membrane, gel, and filter papers were conditioned in the transfer buffer for at least five min. The western blotting technique used was semi-dry transfer, stacking two pieces of filter paper, the membrane and the gel, followed by two filter papers. Gels were transferred at 1000 mA for 50 min. The membrane was then washed with TBST and blocked in 5 % milk in TBST for 30 min. The membrane was shortly washed with TBST and then incubated with primary antibody in TBST at 4 °C on a shaker overnight. (Anti-68 kDa Neurofilament/NF-L antibody (ab9035) rabbit, Abcam ab9035 1:1000; Pgp9.5 rabbit, Proteintech 14730–1-AP, 1:10000, Histone H3, rabbit, Abcam ab1791-100, 1:10000; IL-7 rabbit, PeproTech 500-P57,1:1000, 200 μg/ml stock).

On the next day, the membrane was washed three times with TBST for 10 min and then incubated with secondary antibody (donkey anti-rabbit IgG-HRP, Jackson ImmunoResearch, 711–035-152, 1:10000) for 1 h. The membrane was washed three times for 10 min and then developed with Enhanced chemiluminescence (ECL) Western Blotting-Substrate (Thermo Fisher, 32106) and X-ray films (Fuji super RX). Quantification was performed with ImageJ.

## 3 Results

### 3.1 Culture of sensory neurons in microfluidic chambers yields axonal material of high purity

To investigate transcriptomic alterations after NGF stimulation of DRG sensory neurons in the axonal compartments, we first optimized culture conditions in microfluidic devices containing a central compartment for neuronal cell bodies and two outer compartments for axons (Fig. 1A and B). 200,000 sensory neurons obtained from DRGs of E13 mouse embryos were plated into the central compartment of the chambers (Fig. 1B). An NGF gradient was established to guide the axons into the axonal compartment (Fig. 1B). On DIV 5, densely grown axons were visible in the axonal compartment (Fig. 1C and D). Immunoblot analysis showed that the axonal marker Pgp9.5 but not the nuclear marker histone H3 was present in axonal lysates (Fig. 1E). Immunofluorescence staining revealed that axons in the axonal compartment were positive for Synaptophysin and Munc13a indicating the presence of the synaptic markers and components of the synaptic vesicle release machinery (Supplementary Fig. 1A). Furthermore, cell bodies and axons of compartmentalized DRG neurons were also positive for TrpV1 (Supplementary Fig. 1B). Thus, DRG neurons including nociceptors can be cultured in microfluidic compartments for highly selective axon isolation.

**Fig. 2:**
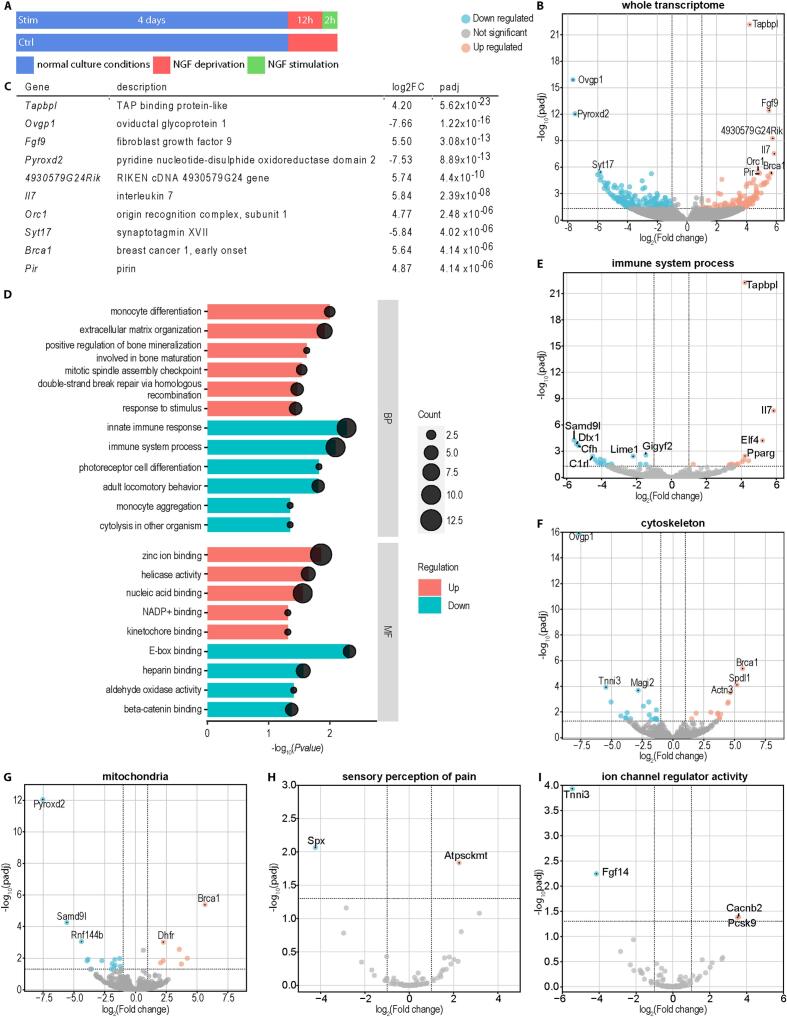
Axonal stimulation with NGF alters transcripts for modulation of the immune system. (A) Schematic representation of the experimental conditions for axonal stimulation with NGF. After DIV 4, axonal NGF was lowered to 2 ng/ml for 12 h, followed by either stimulating with 100 ng/ml NGF (Stim) or maintaining the NGF concentration at 2 ng/ml as a control (Ctrl). (B) Volcano plot depicting the log_2_ fold change and negative log_10_ of the adjusted p (padj) value for all transcripts, comparing stimulated vs. control axons. Cutoffs were set at padj < 0.05 and |log_2_(Fold change)| ≥ 1. (C) Top 10 regulated transcripts according to padj. (D) Top gene ontology terms associated with axonal transcripts altered by NGF stimulation for the categories biological process (BP) and molecular function (MF). Terms enriched for upregulated transcripts are shown in red and for downregulated transcripts in blue. (E-I) Volcano plots for subsets of transcripts belonging to the GO terms ‘immune system process’ (E), ‘cytoskeleton’ (F), ‘mitochondria’ (G), ‘sensory perception of pain’ (H) and ‘ion channel regulator activity’ (I). (For interpretation of the references to colour in this figure legend, the reader is referred to the web version of this article.)

To investigate the effects of NGF stimulation on the axonal transcriptome, compartmentalized DRG neurons were deprived of NGF in the axonal compartment on DIV 4 for 12 h by lowering the axonal concentration of NGF to 2 ng/ml, after which they were stimulated for 2 h with 100 ng/ml NGF (Fig. 2A). NGF was not entirely withdrawn, as complete removal is known to induce stress responses in axons, including the formation of stress granules, degeneration and neuronal death (Maor-Nof et al., 2016). In agreement with this notion, complete removal of NGF led to a significant degeneration of axons after 12 h (Supplementary Fig. 2). As control, we used NGF-deprived neurons without NGF stimulation. RNA was isolated separately from the axonal and somatic compartment and subjected to RNA sequencing (RNA-seq). We first assessed the specificity of the subcellular transcriptome analyses by comparing the axonal and the somatic RNA-seq datasets of the control samples by differential expression analysis (Supplementary Fig. 3A-I). This identified 606 transcripts enriched on the somatic side and 935 transcripts enriched on the axonal side (padj < 0.05 and |log2FoldChange| ≥ 1, Supplementary Table 2). Axon-enriched transcripts were associated with the Gene Ontology (GO) terms ‘ribosome’, ‘mitochondrion’ and ‘synapse’ for the cellular component (CC) category, and ‘translation’, ‘translation at presynapse’, and ‘translation at postsynapse’ for biological process (BP) (Supplementary Fig. 3J). This agrees with observations by others and us that transcripts encoding ribosomal and mitochondrial proteins are enriched in axons of different types of neurons including motoneurons and sensory neurons (Briese et al., 2016, von Kügelgen and Chekulaeva, 2020). The transcripts for ‘ribosome’ and ‘mitochondria’ include those encoding the mitochondrial proteins Cox7c, Cox8a and Trak2 (Supplementary Fig. 3D) and ribosomal proteins like Rps2, Rpsa, Rps12 and Uba52 (Supplementary Fig. 3G). These transcripts are polyadenylated mRNAs that are transported to the axons (Mendonsa et al., 2023). Among mitochondrial transcripts, we also detected mitochondrially encoded rRNAs (mt-Rnr1 and mt-Rnr2). While these results indicate a relative enrichment of the translational machinery and mitochondria in axons compared to somata, we cannot exclude the possibility that their enrichment is due to a lower amount of other RNAs being transported to the axons. In comparison, GO terms associated with soma-enriched transcripts were ‘plasma membrane’, ‘extracellular space’, ‘outer kinetochore’ and ‘chromosome, centromeric region’ for the cellular component category and ‘mitotic spindle assembly checkpoint signaling’, ‘signal transduction’ and ‘monoatomic ion transport’ for the biological process category (Supplementary Fig. 3J). Plasma membrane transcripts include ion channels, receptors, transporters, and adhesion molecules (e.g., cadherins), supporting neuronal excitability, synaptic transmission, and communication. Genes encoding extracellular space proteins such as Wnt2, Tnxb, and Mr1, are involved in extracellular matrix organization and signaling. Wnt2, in particular, suggests ongoing roles for Wnt signaling in synaptic maintenance (Purro et al., 2014). Outer kinetochore and centromeric transcripts, such as those coding for Bub1b, Plk1, Spdl1, Ccnb1, Aurkb, and others, are classical mitotic regulators. However, their expression in neurons likely reflects non-canonical functions in cytoskeletal dynamics, synaptic plasticity, DNA repair, and genome stability (Zhao et al., 2019).

Comparison of the axonally detectable transcripts (Reads Per Kilobase Million (RPKM) > 10 in mean across all axonal Ctrl samples) of DRG neurons to other available datasets showed the presence of all 70 transcripts described as the core neurite transcriptome across neuronal types and species (von Kügelgen and Chekulaeva, 2020). Comparison to the 6,121 axonal transcripts with a FPKM of > 10 detected by Minis et. al., who used explants growing their axons through a membrane, revealed that, of the 17,651 axonal transcripts with an RPKM of > 10 we detected, 5,475 overlapped between the datasets (Supplementary Fig. 4A, (Minis et al., 2014)). These common axonal transcripts were associated with GO terms such as ‘protein binding’, ‘RNA binding’ and ‘protein kinase binding’ for molecular function (MF) and ‘proteasome-mediated ubiquitin-dependent protein catabolic process and protein transport’ for BP (Supplementary Fig. 4B).

### 3.2 Axonal NGF stimulation alters transcripts related to immune response

Comparison of the axonal transcriptomes from NGF-stimulated and unstimulated DRG neurons revealed 183 significantly (padj < 0.05 and |log_2_(Fold change)| ≥ 1, Supplementary Table 3) upregulated and 180 downregulated transcripts (Fig. 2B). However, there was no significant dysregulation of somatic transcripts at 2 h after axonal NGF stimulation, indicating that nuclear gene expression is not altered by axonal NGF treatment during this time period (Supplementary Table 4, Supplementary Fig. 5). Interestingly, the most significantly upregulated transcript encodes for Tapbpl, which is involved in antigen presentation by MHC Class I modulation (Lin et al., 2021), and the most strongly upregulated transcript encodes for the pro-inflammatory IL-7 (Fig. 2B and C). This suggests that one of the earliest axonal transcriptomic changes after NGF stimulation might influence the immune response that is initiated from sensory neurons. The other top upregulated transcripts are *Fgf9*, *Orc1* and *Pirin*. The top downregulated transcripts are *Ovgp1*, *Pyroxd2* and *Syt17* (Fig. 2C). GO term analysis revealed an enrichment of functions related to ‘monocyte differentiation’, ‘extracellular matrix organization’ and ‘double strand break via homologous repair’ among upregulated transcripts, and functions related to ‘innate immune response’, ‘immune system process’ and ‘monocyte aggregation’ among downregulated transcripts (Fig. 2D). Upregulation of the transcript encoding Pirin, a sensor of oxidative stress that limits DNA damage, and the transcripts included in the GO term ‘double strand break via homologous repair’ indicate that mitochondrial maintenance and function might be modulated by NGF stimulation (Hu et al., 2021, Suzukawa et al., 2000). Interestingly, transcripts contained in the GO terms ‘immune system process’, ‘cytoskeleton’ and ‘mitochondria’ were both up- and downregulated (Fig. 2E-G), indicating differential regulation of these pathways. For example, downregulation of the transcript encoding pyridine nucleotide-disulphide oxidoreductase domain 2 (Pyroxd2) might indicate that mitochondrial function is reduced in axons after NGF stimulation (Wang et al., 2019), supporting the notion that mitochondrial dysfunction is associated with pain signaling (van den Ameele et al., 2020). In contrast, Brca1, which is essential for mitophagy and is involved in DNA repair (Chen et al., 2020), is upregulated suggesting a compensatory response to stressed and damaged mitochondria. For the GO term ‘sensory modulation of pain’ only the transcript encoding ATP Synthase C Subunit Lysine N-Methyltransferase (*Atpsckmt*) is upregulated and the transcript for Spexin Hormone (*Spx*) is downregulated (Fig. 2H). For the term ‘ion channel regulator activity’, *Cacnb2* and *Pcsk9* are upregulated and *Tnni3* and *Fgf14* are downregulated (Fig. 2I). *Cacnb2*, encoding calcium voltage-gated channel auxiliary subunit beta 2, can modulate L-type calcium channels, regulating excitability and neuronal activity. Its upregulation has been linked to neuropathic pain (Yu et al., 2019). *Pcsk9*, encoding proprotein convertase subtilisin/kexin Type 9, can degrade the LDLR receptor, dysregulating LDL cholesterol. In DRG neurons, it shows pro-apoptotic properties through reducing ApoER2 and can disturb lipid hemostasis of Schwann cells (Jaafar et al., 2023). Knockout of Pcsk9 might reduce thermal and mechanical pain sensitivity and neuropathy in mice (Jaafar et al., 2024). This effect is accompanied by a reduction in Schwann cells associated with sensory fibers, Remak Fiber axonal swelling, and mitochondrial defects in peripheral nerves. This suggests that an upregulation of Pcsk9 could lead to a sensitization to pain. *Tnni3*, encoding cardiac troponin I, can regulate muscle contractions by blocking actin-myosin interactions depending on the calcium concentration. Its role in neurons is unknown, but it can be dysregulated in postherpetic neuralgia (Qiu et al., 2020). *Fgf14*, encoding the Fibroblast growth factor 14, can modulate NaV channel activity, altering neuronal firing and excitability (Lou et al., 2005). It might also be modulating presynaptic calcium-levels (Yan et al., 2013). These effects, however, seem to be cell type specific (Di Re et al., 2017) and the exact role of Fgf14 in DRGs is not known.

To investigate the specificity of these axonal transcriptome alterations induced by NGF stimulation, we performed RNA-seq analysis on compartmentalized DRG neurons stimulated with ciliary neurotrophic factor (CNTF), which induces pain signaling pathways through STAT3-dependent upregulation of IL-6 (Hu et al., 2020). 191 axonal transcripts were dysregulated by CNTF treatment and downregulated transcripts were associated with GO terms such as ‘synapse organization’, ‘positive regulation of cell adhesion’ and ‘neuron fate commitment’ (Supplementary Table 5, Supplementary Fig. 6). The overlap between the axonal transcripts differentially regulated by NGF and CNTF treatment is very small, with only ten transcripts being significantly dysregulated similarly under both conditions (Supplementary Fig. 7).

### 3.3 Upregulation of axonal *Il7* transcript after NGF stimulation

**Fig. 3:**
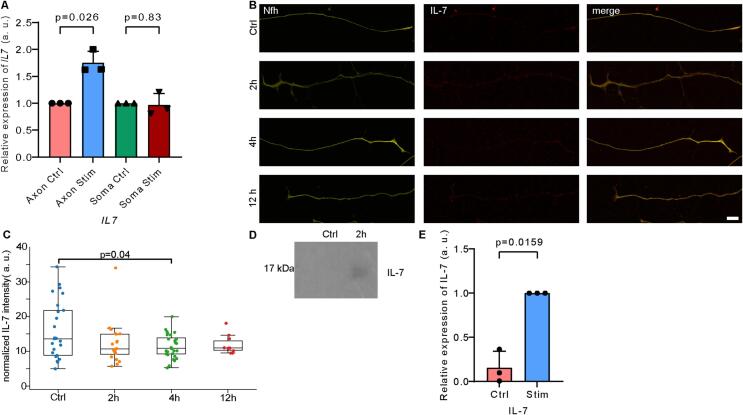
Release of axonal IL-7 upon NGF stimulation. (A) qPCR analysis of *Il7* mRNA levels in soma and axon of unstimulated control and NGF-stimulated DRG neurons. Data are mean ± SD (n = 3). Statistical analysis was performed using a one-sample *t*-test. (B) Immunofluorescence staining of axons after NGF stimulation for Nfh (yellow) and IL-7 (red). Scale bar: 10 μm. (C) Quantification of IL-7 immunosignal intensities. Data are shown as Tukey box plots (n = 10–27 axons from three independent experiments). Statistical analysis was performed using one way ANOVA with Tukey’s post-hoc comparisons. (D) Immunoblot analysis of IL-7 in precipitated axonal supernatants from unstimulated control DRG neurons and DRG neurons stimulated for 2 h with NGF. (E) Quantification of IL-7 levels in axonal supernatants from control and NGF-stimulated (2 h) DRG neurons. Data are mean ± SD (n = 3). Statistical analysis was performed using a one-sample *t*-test. (For interpretation of the references to colour in this figure legend, the reader is referred to the web version of this article.)

According to our data, the *Il7* transcript was one of the top upregulated axonal transcripts after NGF stimulation. To validate this finding, we performed RT-qPCR for *Il7* on axonal and somatic RNA from NGF-stimulated and unstimulated compartmentalized DRG neurons. This showed a 1.75-fold increase in *Il7* transcript levels in axons after 2 h of NGF stimulation, while no change was detectable in the somatic compartment (Fig. 3A). Next, we performed IL-7 immunostaining on stimulated and unstimulated DRG neurons to investigate whether NGF treatment also alters the levels of axonal IL-7 protein. There was a slight reduction in the levels of IL-7 in the growth cones between unstimulated DRG neurons and neurons stimulated with NGF for 4 h (Fig. 3B and C). This points towards the possibility that newly synthesized IL-7 is rapidly secreted from axons following NGF stimulation. To test this possibility, we precipitated total protein from the axonal cell culture supernatant and investigated IL-7 levels by immunoblotting. This revealed a marked increase of IL-7 levels in the supernatant of the axonal compartment by Western blot analysis (Fig. 3D). Quantification of this signal (Fig. 3E) supports the conclusion that high levels of IL-7 are released from axons of sensory neurons after NGF stimulation for 2 h (Fig. 3D and E, one sample *t*-test, p = 0.0159, normality was tested with the Shapiro-Wilk test). Together, this suggests that *Il7* transcripts are rapidly upregulated upon NGF exposure followed by release of IL-7 from axons into the extracellular environment.

### 3.4 Assembly of ribosomal subunits in axonal growth cones is not increased by NGF stimulation

Our data indicate that IL-7 is rapidly synthesized in axons upon NGF treatment. This might involve rapid assembly of ribosomal subunits for translational activation. To address this possibility, we performed immunostaining of NGF-stimulated and unstimulated DRG neurons for the ribosomal proteins Rpl24 and Rps6, which are components of the 60S and 40S ribosomal subunits. As the two ribosomal subunits assemble, they move together spatially within a range of 200–300 Å, which can be visualized using Structured Illumination Microscopy (SIM), as previously demonstrated (Deng et al., 2021). Statistical analysis was performed using Kruskal-Wallis test and subsequent pairwise comparisons (n = 60–80 growth cones from three independent experiments). Co-staining and subsequent colocalization analysis showed that co-localization of Rpl24 and Rps6 was significantly reduced after 1 min (p = 0.025) NGF stimulation but not after 10 or 30 min. (Supplementary Fig. 8). This indicates that mechanisms other than enhanced ribosome assembly such as re-activation of stalled polysomes contribute to enhanced IL-7 synthesis upon NGF stimulation (Anadolu et al., 2023, Graber et al., 2013).

### 3.5 Axonal NGF stimulation causes a transient increase in the surface localization of the TrkB receptor

**Fig. 4:**
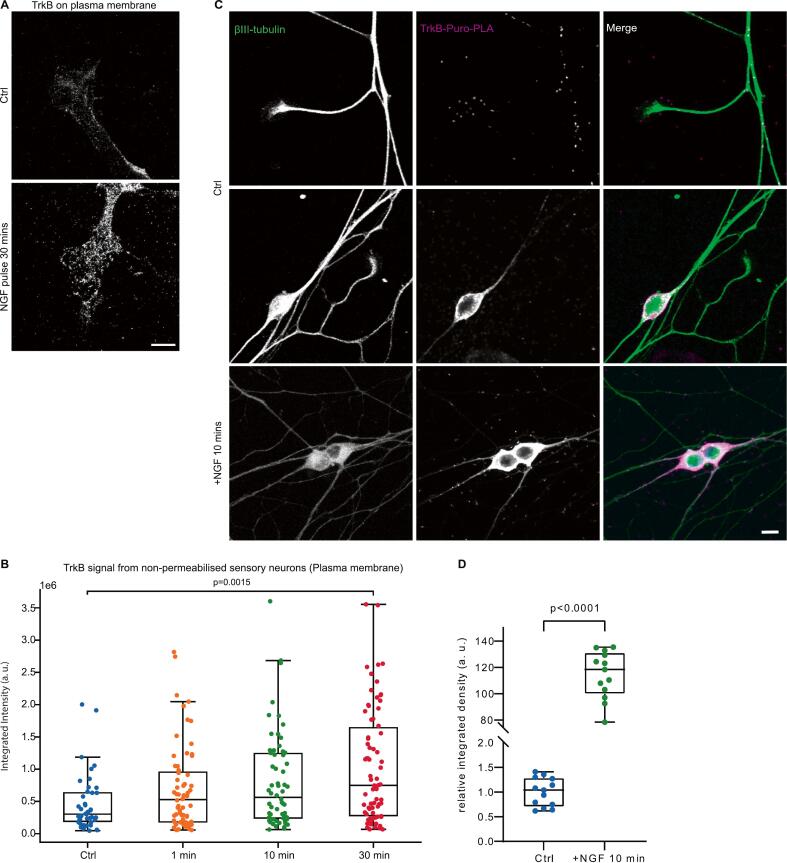
Enhanced synthesis and surface translocation of TrkB upon NGF stimulation. (A) Presence of TrkB at the plasma membrane visualized by super-resolution microscopy (SIM) in growth cones of compartmentalized microfluidic chambers. Scale bar: 5 µm. (B) Quantification of TrkB signal on the plasma membrane. Data are shown as Tukey box plots (n = 43–74 growth cones from three independent experiments). Statistical analysis was performed using Kruskal-Wallis test and subsequent pairwise comparisons. (C) TrkB-Puro-PLA signal in cultured control DRG neurons and DRG neurons stimulated with NGF for 10 min. Scale bar: 5 μm. (D) Quantification of the Puro-PLA signal intensities in somata. Data are shown as Tukey box plots (n = 14 cells). Statistical analysis was performed using Mann-Whitney Test.

While NGF signals via binding to the TrkA receptor it is possible that NGF stimulation might also lead to secondary signaling via other neurotrophic factors such as brain-derived neurotrophic factor (BDNF) (Michael et al., 1997). To investigate whether auto- or paracrine BDNF-TrkB signaling is induced by NGF stimulation, the axonal compartment of DIV 3 sensory neurons grown in microfluidic chambers was treated with NGF followed by a cell-surface TrkB localization assay (Hennlein et al., 2023) (Fig. 4A). This revealed a steady increase in the surface TrkB localization at the growth cones after NGF stimulation over the course of 30 min (Fig. 4B Kruskal-Wallis test and subsequent Pairwise comparisons, p adj. = 0.0015). This indicates that NGF exposure, in addition to modulating the axonal transcriptome, induces BDNF-TrkB signaling via regulating its plasma membrane localization.

Since the transcripts encoding Trk receptors were not altered significantly by NGF stimulation in our RNA-seq data, we questioned whether there is a change in the surface presentation of TrkB receptors due to enhanced de novo synthesis of TrkB protein by NGF. Therefore, we investigated whether a pulse of NGF affects the intracellular production of TrkB at different subcellular sites. For this purpose, we performed a puromycin labeling proximity ligation assay (Puro-PLA) on NGF-stimulated and unstimulated DRG neurons to label nascent TrkB. In this assay, puromycin is incorporated into actively translated polypeptides. As a result, PLA with antibodies against the N-terminus of TrkB and puromycin can aid the visualization of newly synthesized TrkB receptors (tom Dieck et al., 2015). The signal corresponding to TrkB-Puro-PLA was observed mainly near the cell soma (Fig. 4C). We observed a marked increase in this Puro-PLA signal near the soma after 10 mins of NGF stimulation concurrent to the Puromycin treatment (Fig. 4D, Mann-Whitney Test, p < 0.0001). PLA Signal from the axons and growth cones was observed sporadically and these areas were therefore excluded from the analysis.

## 4 Discussion

NGF is released after injury and under inflammatory conditions from macrophages, fibroblasts and other types of nonneuronal cells (Barker et al., 2020). NGF then binds to the TrkA receptor and the pan-neurotrophin receptor p75NTR on sensory neurons and elicits nuclear responses after retrograde transport to the soma, but also local responses at the axonal sites (Campenot, 1994). These local responses include rapid effects on the cytoskeleton and modulation of ion channels such as TrpV1 via local PI3K and Src kinase signaling (Ketschek et al., 2015, Zhang et al., 2005). These rapid local effects are highly relevant for the functionality of nociceptors. In many types of neurons, neurotrophin signaling has strong effects on local protein production (Lin and Holt, 2008). In axons and presynaptic terminals, neurotrophin signaling also leads to rapid assembly of ribosomes, both polysomal particles and also ribosomes that are associated with the axonal tubular endoplasmic reticulum (Deng et al., 2021). These effects are induced by BDNF in motoneurons and other types of neurons from the central nervous system. These mechanisms create the prerequisites also for both rapid axonal changes that contribute to acute pain sensitization, as well as the long-term alterations responsible for chronic pain.

However, the dynamic regulation of transcripts in axons of sensory neurons involved in pain sensitization and chronic pain induction has so far not been investigated in detail. In particular, we study the dynamic changes in the axonal transcriptomic profile after local stimulation with NGF and the induced effect on localized signaling.

In order to study local effects of NGF on axonal transcript distribution, protein synthesis and TrkB distribution, we optimized techniques for microfluidic cultures of embryonic DRG neurons. The use of embryonic rather than adult DRG neurons allowed us to obtain a sufficient number of neurons to harvest axonal RNA for RNA-seq analysis following axonal NGF stimulation. While this might differ from adult DRG neurons, it is possible that the neurons further mature in culture due to presence of the neurotrophins NGF, BDNF, CNTF and GDNF (Huang and Reichardt, 2001). Previous studies have shown that E13 sensory neurons already exhibit many electrophysiological properties that are characteristic of functional adult sensory neurons (Koltzenburg and Lewin, 1997). Interestingly, most of the classical transcripts involved in pain signaling were not altered. This is consistent in models of induced peripheral inflammation or intrathecal NGF treatment, where *Trpv1* mRNA levels in DRGs are not significantly altered, but the TrpV1 protein levels for the same are increased (Ji et al., 2002, Voilley et al., 2001). Our data revealed a strong modulation of transcripts, in particular elevated levels of transcripts related to immune responses shortly after stimulation. Of note, transcripts that potentially modify antigen presentation and immune cell activity, such as *Tapbpl*, *Fgf9* and *Il7* are among the top hits (Chen et al., 2021, Deng et al., 2018, Lin et al., 2021). This indicates that one of the earliest axonal responses to NGF stimulation regulates the neuro-immune axis.

In the last few years, the interaction between immune cells and neurons and their importance for the regulation and chronification of pain has come into focus (Brack et al., 2004, Lacagnina et al., 2024, Marek-Jozefowicz et al., 2023). For example, the dermal macrophages can set a baseline of pain sensitivity through the release of NGF (Tanaka et al., 2023). The neurons themselves can be activated and sensitized by different signaling molecules secreted by immune cells. These normally are cytokines, bradykinin, prostaglandins, tumor necrosis factor and neurotrophic factors (Basbaum et al., 2009). Vice versa, neurons can regulate the immune cells and cause neurogenic inflammation through the release of substance P, CGRP and interleukins like IL-6 (Marek-Jozefowicz et al., 2023). In an acute injury, the concentration of NGF in the injured tissue is increased due to its release from nonneuronal cells like macrophages (Lindholm et al., 1987), acting upon other immune cells and the neurons themselves. The NGF uptake by sensory neurons through binding to TrkA leads to local modulation of sensitivity by influencing local translation and modulation of channels and receptors (Barker et al., 2020).

For the immune response, one of the most strongly upregulated transcripts is *Il7*. IL-7 itself is pro-inflammatory and signals through the JAK1, JAK3, and PI3K pathways, which leads to STAT5 phosphorylation (Chen et al., 2021). IL-7 activation can lead to a transcriptional shift in the activated cells. It has also been implicated in autoimmune diseases like multiple sclerosis and rheumatoid arthritis (Meyer et al., 2022). IL-7 activates various immune cells, including CD4^+^, CD8^+^ and dendritic cells (Choi et al., 2016). Furthermore, IL-7 attracts circulating immune cells such as T-cells, B-cells and NK cells and promotes their survival. This is achieved through upregulation of Bcl-2 and Mcl-1, which inhibit apoptosis (Chen et al., 2021). IL-7 also influences the maturation and development of dendritic cells, driving macrophage differentiation to increase the expression of major histocompatibility complex Class I and II, CD21, CD86, and CD40 (Li et al., 2000). Moreover, IL-7 can remodel macrophages into inflammatory M1 cells. The IL-7 receptor expression is upregulated by lipopolysaccharide, Interferon-γ and TNFα. Local expression of IL-7 can induce arthritis (Chen et al., 2021, Kim et al., 2020). Our finding of increased axonal *Il7* transcript levels and IL-7 release from axons after NGF stimulation of sensory neurons supports the notion that DRG neurons locally and rapidly modulate immune processes, in particular in macrophages and immune presenting cells. Since NGF is involved in pain induction, this suggests that IL-7 that is released from neurons after NGF induction might be a central component of pain network signaling that involves the interplay between sensory neurons and immune cells in the periphery. While the exact mechanism for IL-7-release is unknown, it might be similar to other Interleukins like IL-6, and thus be influenced by TrkA in the same way. TrkA activates both the PI3K and the PLCγ pathways (Denk et al., 2017). PI3K can remodel actin and activate release through the phosphorylation of proteins of the SNARE complex through Act (Brose et al., 1992, Catterall, 1999). PLCγ can increase intracellular calcium concentration and activate Synaptotagmin. Both pathways are involved in vesicle release of neuropeptides and under inflammatory conditions, IL-6 has been shown to be released in response to increased calcium levels and PKC activation (Cadman et al., 1994, Qiu et al., 1995). The release of IL-7 from the neurons can shift the surrounding immune cells to an inflammatory state, contributing to the establishment of an inflammatory milieu (Chen et al., 2021). These activated immune cells release mediators like IL-1β and TNF-α. This in turn can sensitize not only the neuron that released IL-7, but also the surrounding neurons. A possible mechanism on nociception has been described in a companion paper by Biesold et al in this issue.

Apart from potential local remodeling and immune cell recruitment, NGF is also known to promote long-lasting shifts in excitability of nociceptors. This appears to be a central mechanism in pain chronification. Injection of NGF into neonatal rats has a persisting effect on mechanical and heat hyperalgesia. This effect seems to be due to a permanent NGF-mediated sensitization of Aδ nociceptors to mechanical stimuli (Lewin and Mendell, 1993). Mechanical hyperalgesia after NGF injection in neonatal rats is caused by central changes in spinal cord wiring of these afferents (Lewin et al., 1992b) whereas the heat hyperalgesia was proposed to be due to sensitization of peripheral receptors (Lewin et al., 1992a). The molecular mechanisms underlying this long-lasting sensitization are still not fully understood. The potentiation of synaptic connections between pain- and mechanoconducting neurons and spinal dorsal horn neurons seems to involve BDNF (Thompson et al., 1999).

BDNF itself is dynamically regulated after nerve injury. BDNF is detectable in dorsal horn as early as 24 h post-injury, with subsequent rise in the BDNF protein levels (Thakkar and Acevedo, 2023). While these levels revert to normal typically within 4–6 weeks after the injury, persistently high levels of BDNF expression have been associated with aberrant healing and or sustained pain signaling. This temporally and spatially controlled expression pattern implicates BDNF in adaptive recovery, or maladaptive plasticity associated with chronic pain (Fukuoka et al., 2001, Lin et al., 2011).

Given the primary effects of NGF, we also investigated the potential contribution of BDNF/TrkB signaling to longer-term alterations in sensory neuron excitability. In contrast to NGF, BDNF is expressed within sensory neurons, and released from central terminals in the spinal cord to act on postsynaptic neurons (Thompson et al., 1999). However, BDNF also seems to be present in peripheral axonal branches, raising the question whether it could act locally on TrkB receptors in nociceptors and induce long-lasting changes, similar to those observed in neurons of the central nervous system (Park and Poo, 2013).

We have focused our attention on BDNF/TrkB signaling, since BDNF/TrkB signaling is known for its role in modulation of long-lasting alterations in neural circuits (Mizuno et al., 2003). BDNF/TrkB signaling is a potent inducer of local translation at axon terminals, activating MAPK, phosphatidylinositol 3-kinase and the mTOR pathways (Numakawa et al., 2010). In the periphery, BDNF is primarily expressed by sensory neurons (Yu et al., 2020), though reports indicate also minor expression of BDNF from other cell types such as microglia during neuropathic pain (Coull et al., 2005). Thus, BDNF/TrkB-mediated activation of the PI3K pathway in axons of sensory neurons could contribute both to the regulation of local protein synthesis, as observed in motoneurons (Deng et al., 2021), and the local modulation and sensitization of TrpV1 (Zhang et al., 2005), possibly with long-lasting effects when NGF treatment leads to chronic activation of the BDNF/TrkB signaling loop. Upon binding of BDNF to the TrkB receptor, the receptor ligand complex is endocytosed in a clathrin dependent manner (Philippidou et al., 2011, Valdez et al., 2005, Zheng et al., 2008). BDNF/TrkB signaling is modulated by NGF exposure in sensory neurons (Karchewski et al., 2002). Extracellular BDNF can also regulate the TrkB surface expression in neurons, thus acting as a self-regulating mechanism (Haapasalo et al., 2002). However, this system can be adapted according to local physiological needs in the presence of parallel signaling through different molecular complexes, especially other neurotrophin-receptor complexes such as NGF-TrkA. Therefore, pain can involve persistence of certain molecular states, maintained by signaling of complexes that can act in an autocrine manner and facilitate the maintenance of a certain transcription profile, leading to changes in neuronal physiology and therefore firing patterns.

BDNF also plays a complex role in the development of neuropathic pain (Alles et al., 2021, Biggs et al., 2010, Sikandar et al., 2018). In male rats, proBDNF/p75NTR signaling has been shown to be crucial for the onset and maintenance of neuropathic pain (Li et al., 2023). BDNF-TrkB signaling also affects the chloride gradient in neurons, switching the inhibitory effect of GABA to excitation (Dai and Ma, 2014). This has been shown to further increase excitability and pain perception. Consequently, there have been attempts to decrease the BDNF-dependent chloride channel-induced hyperexcitability through the use of chloride ion channel modulators (Price et al., 2009). Moreover, BDNF promotes the release of pro-inflammatory cytokines from astrocytes, adding to the heightened pain response (Smith, 2024).

The increased synthesis and membrane insertion of TrkB upon NGF exposure that we observed in our study might further sensitize neurons to respond effectively to injury (Andreska et al., 2020). Future studies on in-depth ribosomal profiling at different time points after NGF exposure could give deeper insights into the repertoire of proteins synthesized locally in axons under conditions of pain sensitization and/or chronic pain induction.

In summary, our findings reveal that NGF stimulation modulates the axonal transcriptome towards neuro-immune signaling activation while pain-related transcripts were only modestly affected. The strong modulation and release of IL-7 emphasizes the role of sensory neurons in modulating the local immune processes shortly after injury. This could be crucial for the initiation and maintenance of inflammation and pain. Besides the transcriptional modulation, there were also influences on translation and localization of TrkB. The interplay between NGF-TrkA and BDNF-TrkB signaling pathways could play an important role in the neuronal adaptation and sensitization during injury. These findings align with previous studies that showed a pivotal role of neutrophins in regulating the transition from acute to chronic pain states through modulating networks in both neurons and immune cells.

## CRediT authorship contribution statement

**Maximilian Koch:** Writing – review & editing, Writing – original draft, Visualization, Methodology, Investigation, Formal analysis, Data curation, Conceptualization. **Manas Kshirsagar:** Writing – review & editing, Writing – original draft, Visualization, Methodology, Investigation, Formal analysis, Data curation, Conceptualization. **Ankita Rawat:** Investigation. **Abdolhossein Zare:** Methodology. **Felicitas Schlott:** Visualization, Formal analysis. **Thorsten Bischler:** Formal analysis, Data curation. **Panagiota Arampatzi:** Formal analysis, Data curation. **Michael Briese:** Writing – review & editing, Writing – original draft, Supervision, Project administration, Funding acquisition, Conceptualization. **Michael Sendtner:** Writing – review & editing, Writing – original draft, Supervision, Project administration, Funding acquisition, Conceptualization.

## Declaration of competing interest

The authors declare that they have no known competing financial interests or personal relationships that could have appeared to influence the work reported in this paper.

## Data Availability

The RNA-seq data have been deposited at Gene Expression Omnibus (GEO) under accession number GSE292014.
