# Supplementary figures and images for: NGF stimulation alters the transcriptome and surface TrkB expression in axons of dorsal root ganglion neurons
Source: Neurobiol Pain. 2025 Aug 5;18:100194. doi: 10.1016/j.ynpai.2025.100194 (PMC12355597; doi:10.1016/j.ynpai.2025.100194)

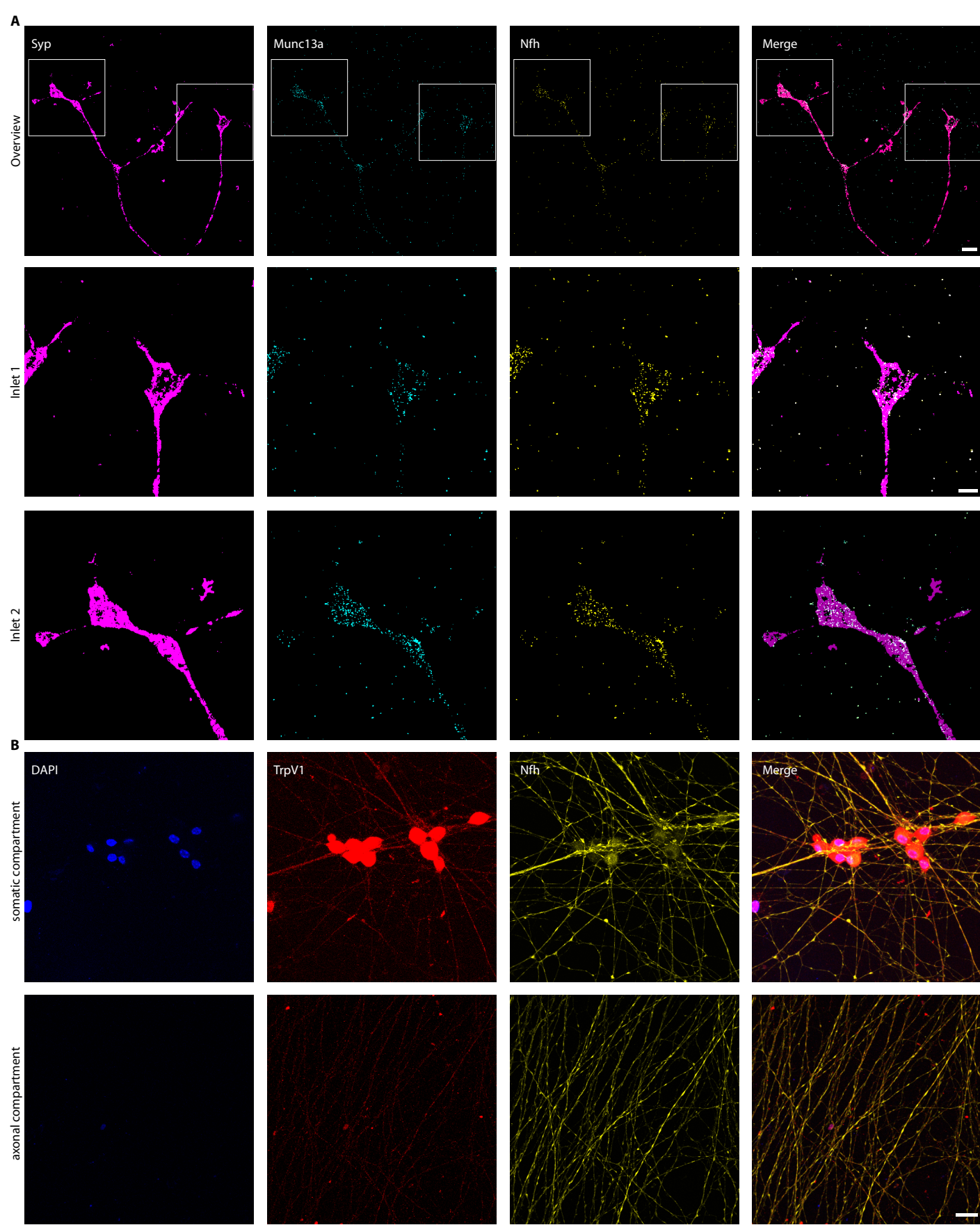

Supplement: Supplementary Data 1 [file mmc1.pdf]

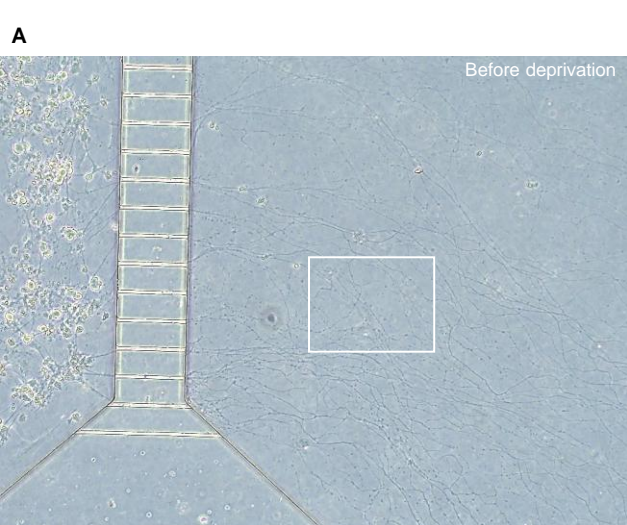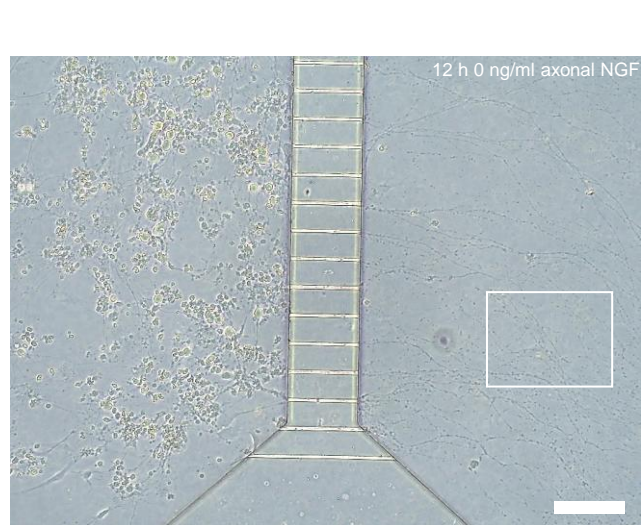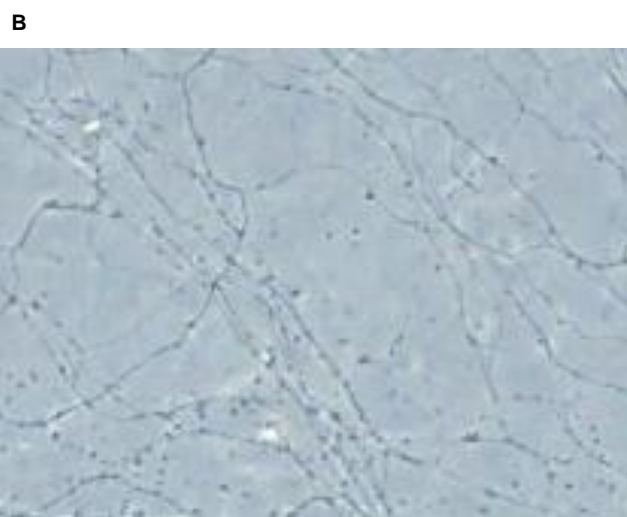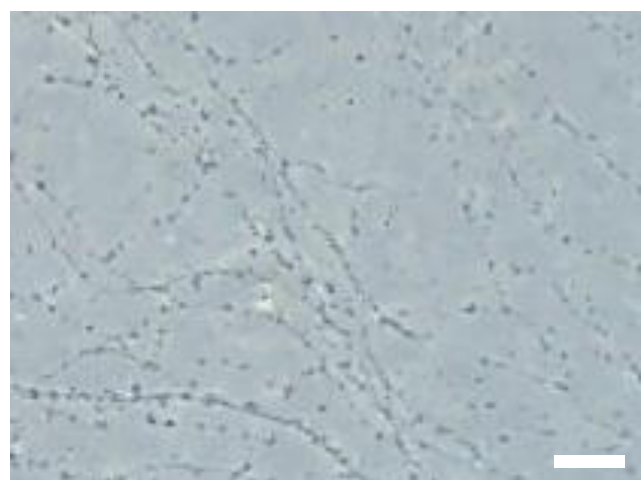

Supplement: Supplementary Data 2 [file mmc2.pdf]

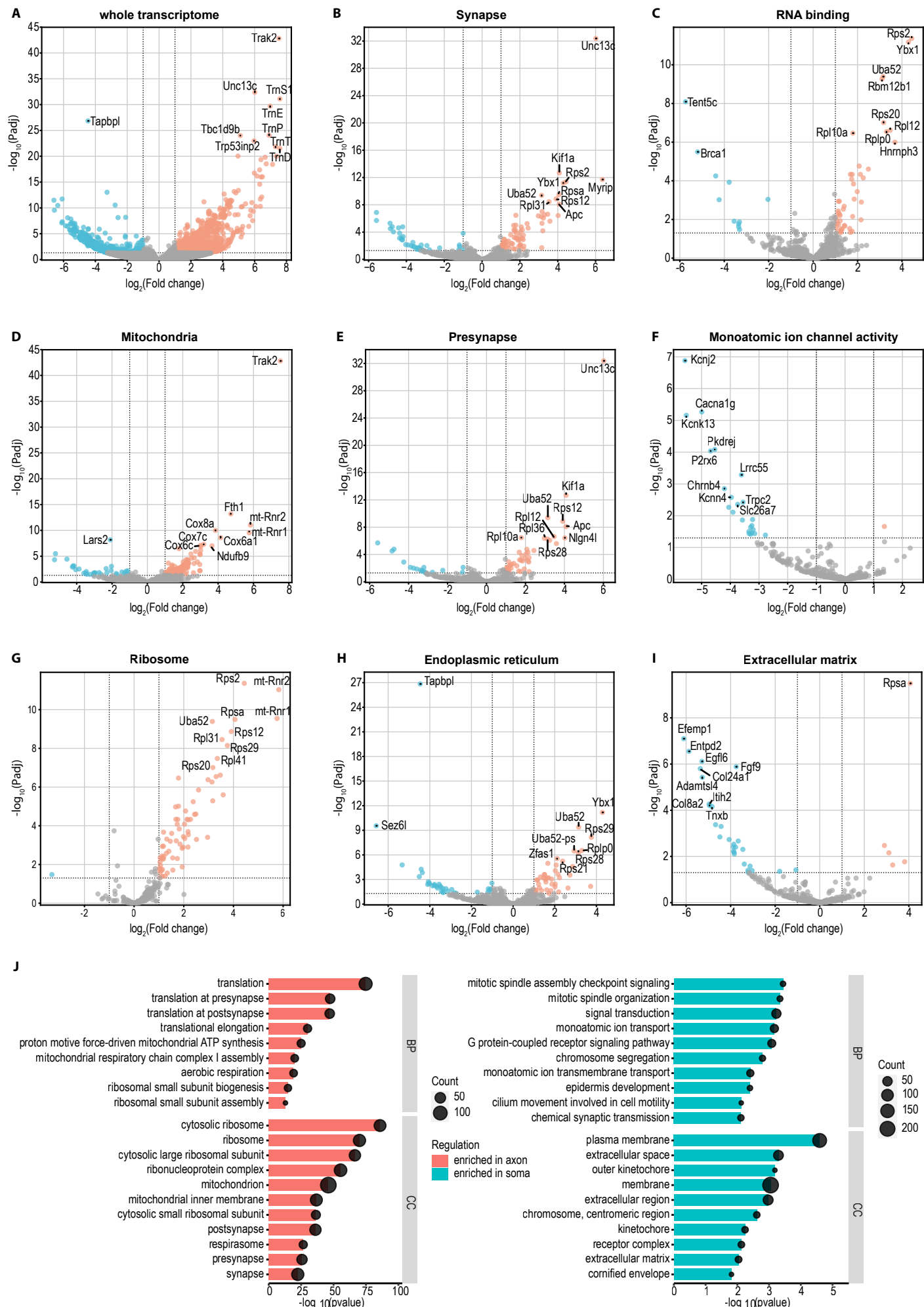

Supplement: Supplementary Data 3 [file mmc3.pdf]

A

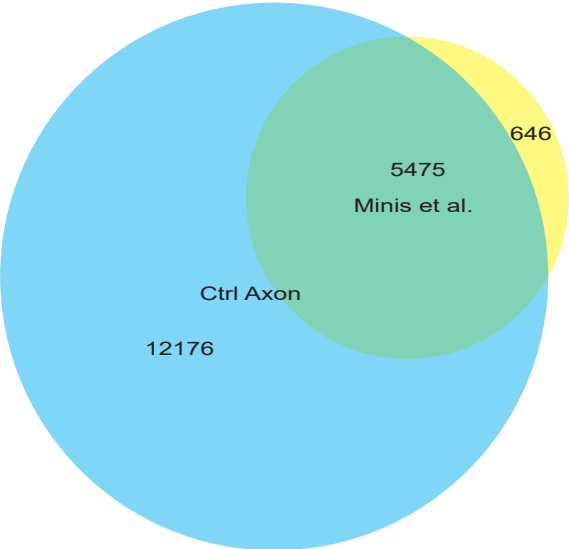

B

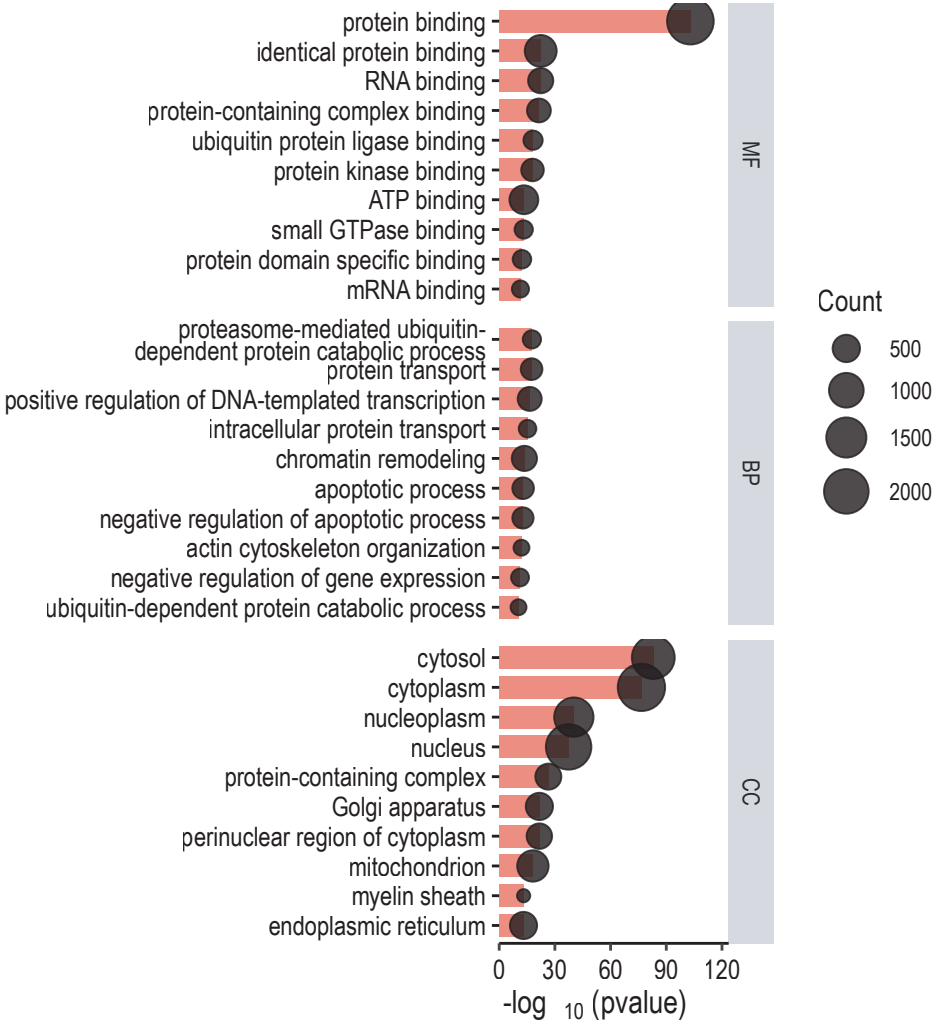

Supplement: Supplementary Data 4 [file mmc4.pdf]

Down regulated (0)

● Not sig (32196)

Up regulated (0)

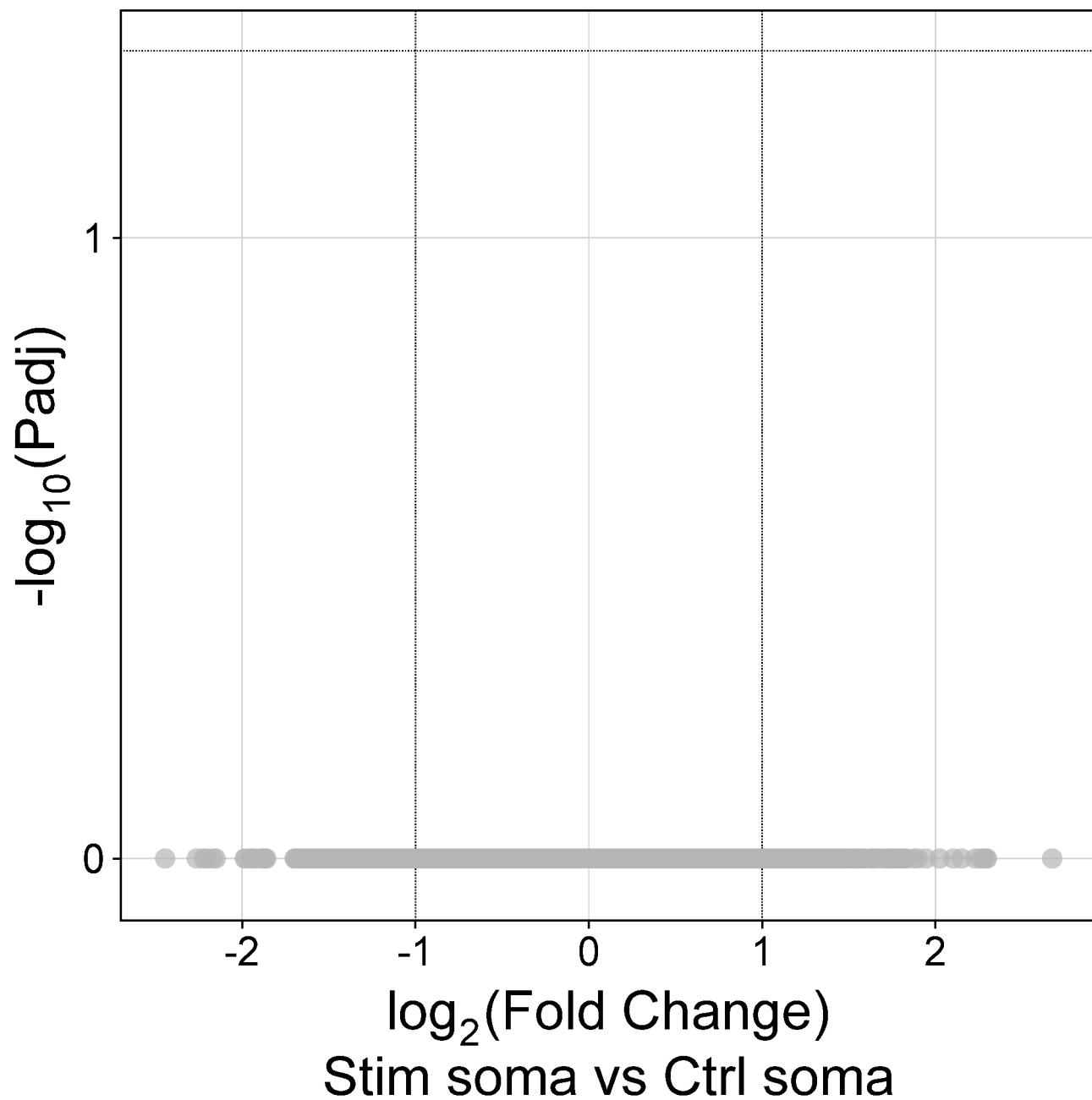

Supplement: Supplementary Data 5 [file mmc5.pdf]

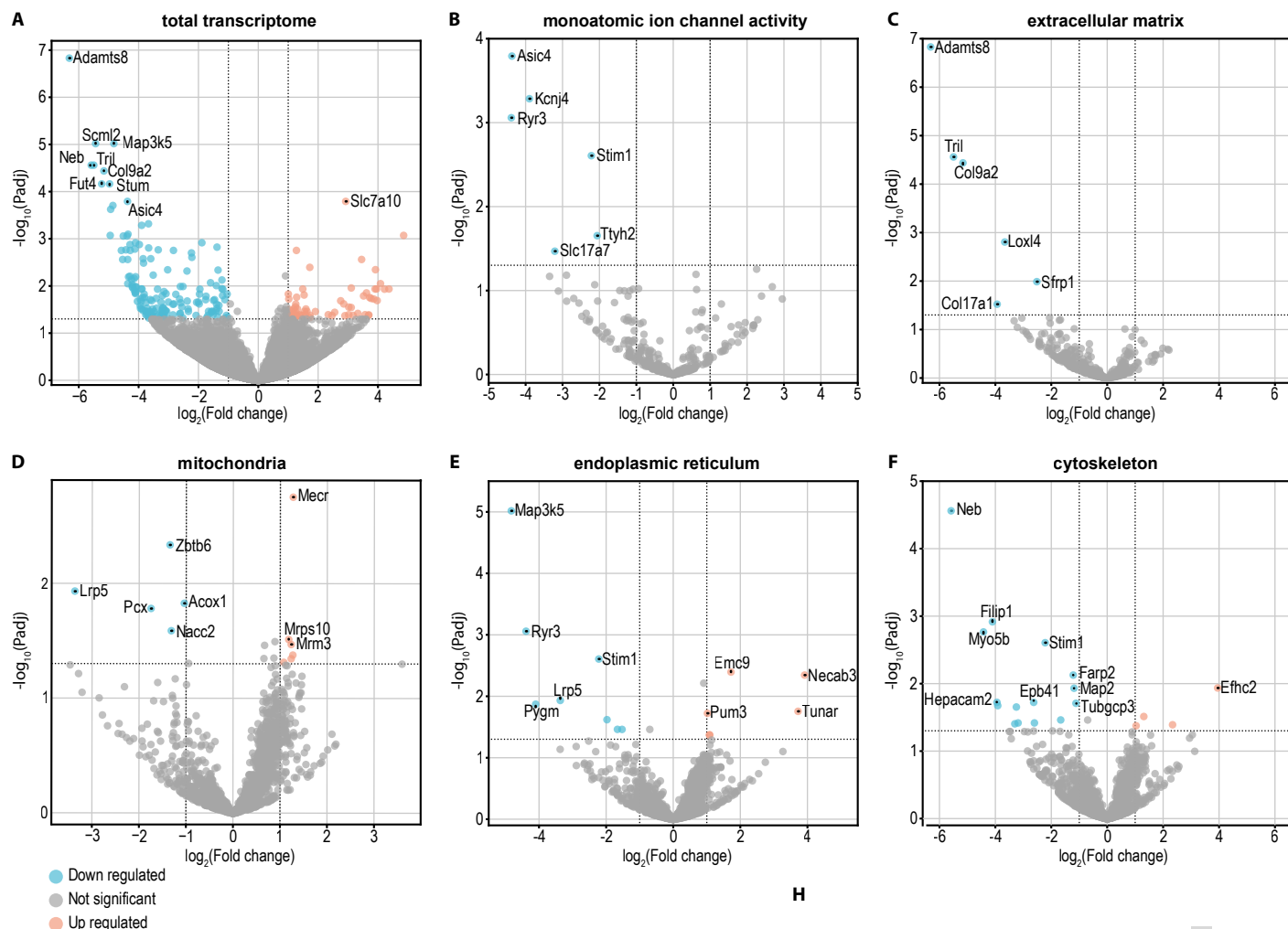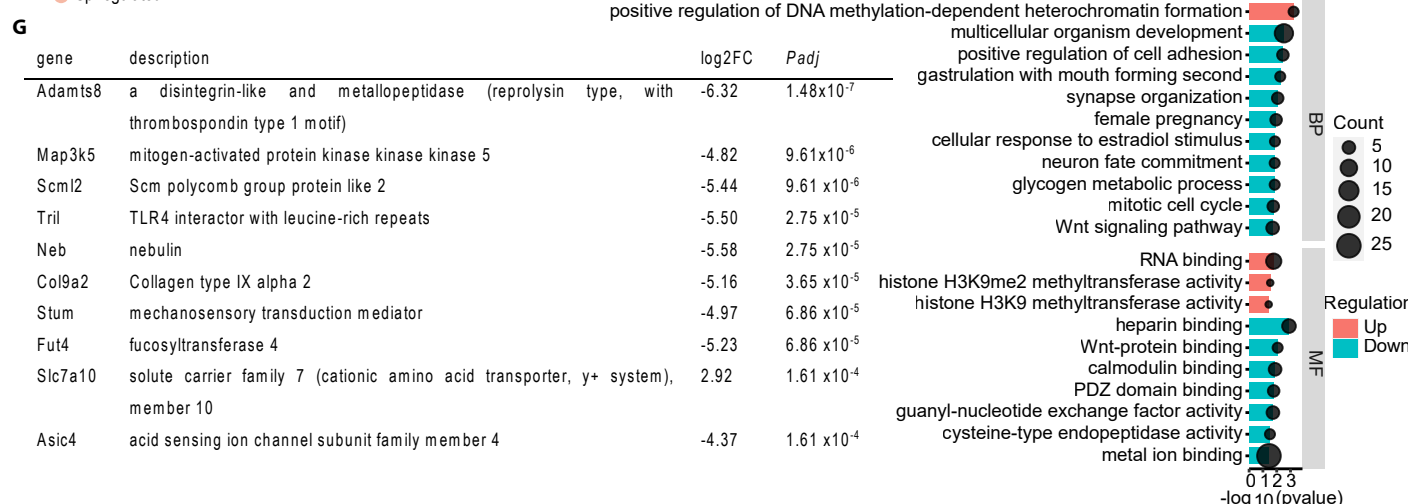

Supplement: Supplementary Data 6 [file mmc6.pdf]

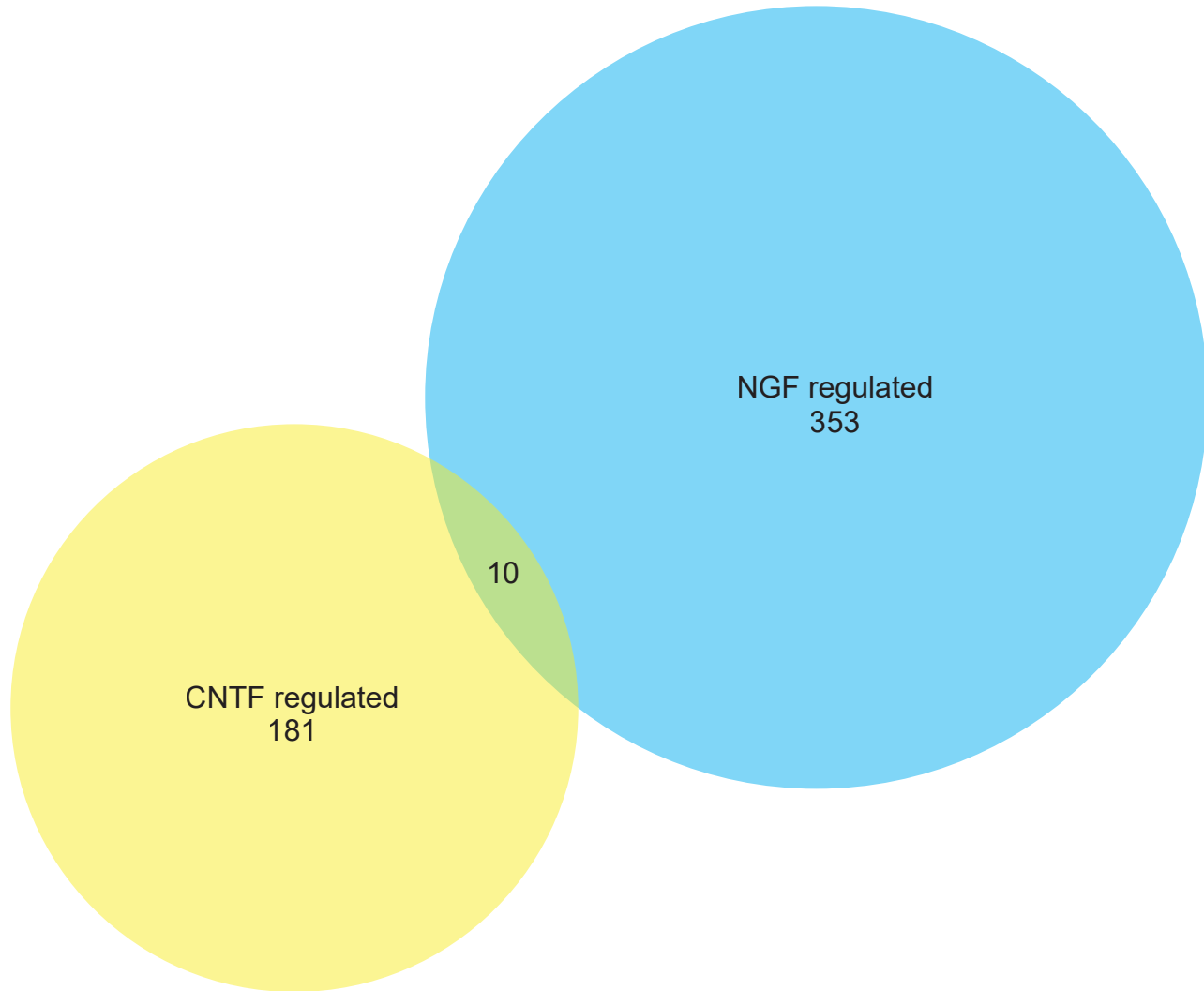

Supplement: Supplementary Data 7 [file mmc7.pdf]

**A**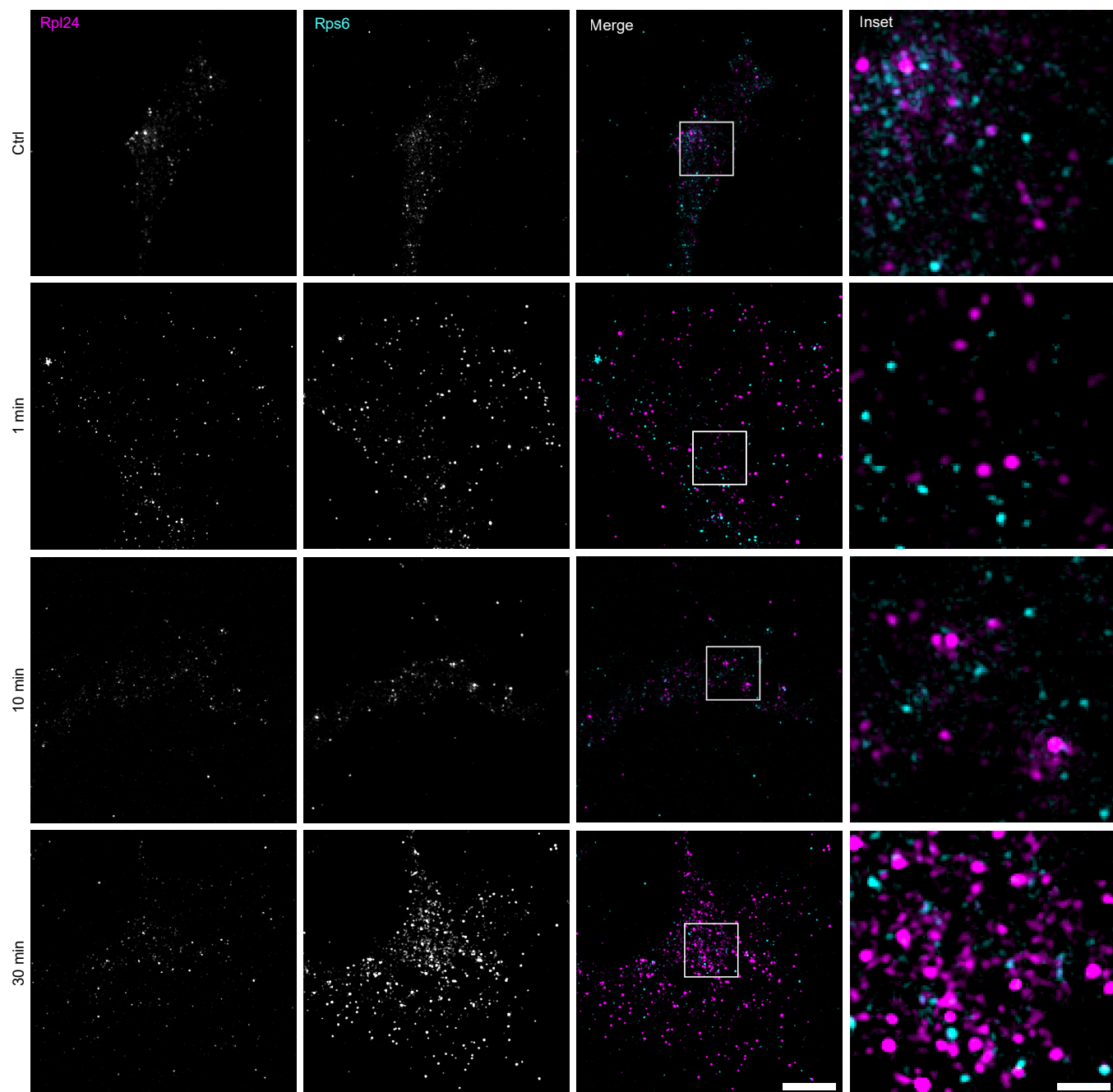**B**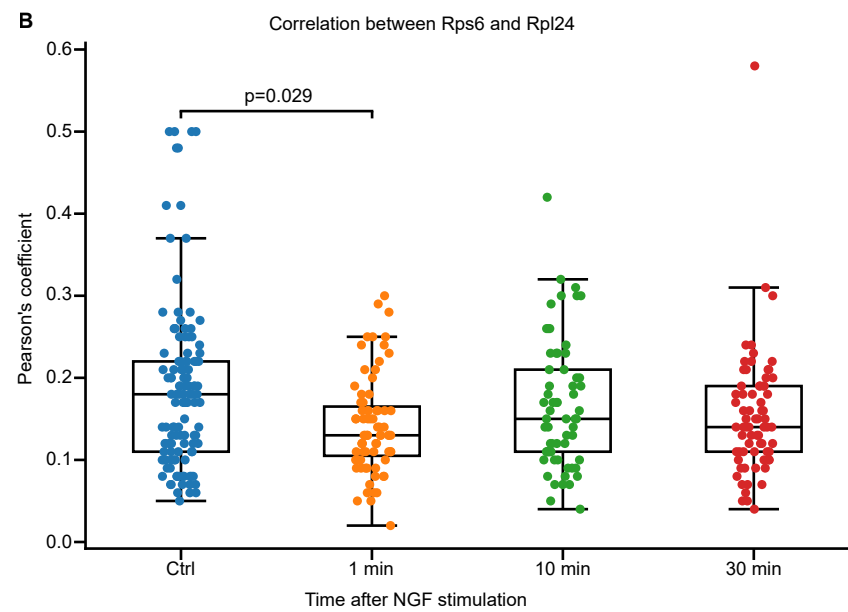

Supplement: Supplementary Data 8 [file mmc8.pdf]
